# Parliament and the Professions

**Published:** 1919-08-09

**Authors:** 


### PARLIAMENT AND THE PROFESSIONS.

### Artificial Appliances for Officers and Men.

Sir L. Worthington-Evans announced that the cost of repairs and renewals of artificial limbs and other appliances will in future for officers, as for men, be borne by the State. This decision has been extended to artificial appliances other than limbs. The serving officer must bear the cost of renewals and repairs until retired, but on his retirement the Ministry of Pensions will undertake such expenses when necessary from fair wear and tear.

### Physical Condition of Recruits.

Col. Sir James Craig informed Major Farquharson that the whole of the medical and statistical staff of the Ministry of National Service were transferred to the Ministry of Pensions on April 1 last, and that part of that staff have been for some time engaged on the work of collating and adopting the scientific data of documents in connection with the physical condition of Army recruits.

### Isle of Man Dental Register.

In answer to a question to the Minister of Health by Mr. Seddon, it was stated that the Dentists' Register is now common to the United Kingdom and the Isle of Man. By the Isle of Man Dental Act, 1908, provision was also made for a " supplemental register " of persons in practice in the island on January 1, 1908, and such persons can only practice locally; following upon the report of the Dentists' Committee the position of such persons will be carefully considered.

### National Insurance.

Dr. Addison stated that in September 1917 special arrangements were made as to the provision of medical benefit for men invalided from war service; the estimated number of men admitted to this benefit under those arrangements is 318,000. There are, however, a number of men who were eligible for medical benefit before September 1917, but they became merged in the civil population.

### Naval Nursing Sisters.

In answer to a question by Sir Bertram Falle, Dr. MacNamara stated that the rates of pay to reserve nursing sisters under Queen Alexandra's Royal Naval Nursing Service were recently revised, and the rates for nursing sisters on active service are at present under consideration. No war bonus is payable to nurses, as they are in receipt of free victualling, and the larger part of their uniform is provided at public expense.

